# US-derived Pediatric Kidney Length and Volume Percentiles by Age: A Big Data Approach

**DOI:** 10.1148/ryai.250056

**Published:** 2025-11-19

**Authors:** Bernarda Viteri, Tatiana Morales-Tisnés, Joey Logan, Jarcy Zee, Summer Kaplan, Susan J. Back, Erum A. Hartung, Madhura Pradhan, Lisa Guay-Woodford, Susan L. Furth, Sandra Amaral, Gregory E. Tasian, Hansel J. Otero

**Affiliations:** ^1^Division of Nephrology, Department of Pediatrics, Children’s Hospital of Philadelphia, 3401 Civic Center Blvd, Philadelphia, PA 19104; ^2^Perelman School of Medicine, University of Pennsylvania, Philadelphia, Pa; ^3^Department of Biomedical and Health Informatics, Children’s Hospital of Philadelphia, Philadelphia, Pa; ^4^Children’s Hospital of Philadelphia Research Institute, Philadelphia, Pa; ^5^Department of Biostatistics, Epidemiology, and Informatics, Perelman School of Medicine, University of Pennsylvania, Philadelphia, Pa; ^6^Department of Surgery, Division of Urology, Children’s Hospital of Philadelphia, Philadelphia, Pa; ^7^Department of Radiology, Children’s Hospital of Philadelphia, Philadelphia, Pa

**Keywords:** Kidney, Natural Language Processing, Pediatrics, Ultrasound

## Abstract

**Purpose:**

To calculate new pediatric age-specific normative values and percentiles for kidney length and volume through the use of a natural language processing (NLP) model.

**Materials and Methods:**

In this cross-sectional study, 24 664 US reports from 18 769 children (birth to 18 years) conducted between January 2012 and December 2022 at a tertiary children’s hospital in the northeastern United States were analyzed with an NLP model. Anthropometric data from 12 595 children were used to evaluate the effect of sex and body measurements on kidney length and volume through age-adjusted quantile regression models. Age-related percentiles were established after calibration, using the lambda-mu-sigma (LMS) method by age (year), with detailed subcategories for children younger than 1 year. Volume percentiles by body surface area were also generated using the LMS method.

**Results:**

A total of 24 664 reports from 18 769 children were included (median age, 7 years [IQR, 11 years]; 10 134 female children). Normative value analysis showed that kidney growth was more pronounced in the 1st year of life (1.8-cm increase in length and 16.9-cm^3^ increase in volume). The large sample size resulted in standard errors that were 10%–30% less than previous normative values. Quantile regression models showed that body surface area was a better predictor of kidney volume than was age (R^1^ = 0.57 [*P* < .001] vs 0.48 [*P* < .001]).

**Conclusion:**

New LMS percentiles for kidney size were established using data from a large pediatric sample.

**Keywords:** Kidney, Natural Language Processing, Pediatrics, Ultrasound

*Supplemental material is available for this article.*

© The Author(s) 2025. Published by the Radiological Society of North America under a CC BY 4.0 license

See also the commentary by Sihlahla in this issue.

## Graphical abstract

**Figure:**
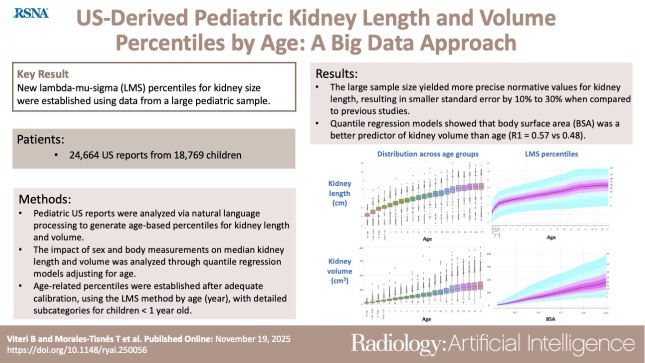

**Summary**

A large dataset of pediatric US measurements and natural language processing was used to calculate kidney length and volume percentiles, resulting in increased normative value precision and highlighting growth patterns.

**Key Points**

■ A total of 24 664 US reports from 18 769 children were analyzed using natural language processing to generate age-based percentiles for kidney length and volume, highlighting key growth patterns in early childhood.
■ The large sample size yielded more precise normative values for kidney length, resulting in smaller standard error by 10%–30% when compared with previous studies.
■ Analysis of anthropometric measurements from 15 891 US reports via quantile regression models revealed that body surface area was a better predictor of kidney volume than was age (R^1^ = 0.57 [*P* < .001] vs 0.48 [*P* < .001]).

## Introduction

Kidney size measured with US is a practical anatomic marker used by clinicians to screen and diagnose kidney disease in children, as well as to assess interval growth and guide medical decisions (1,2). Measurements of kidney volume are more accurate, but kidney length, measured along the long axis (pole to pole), is most commonly used because it is less susceptible to interobserver variability (3,4). Kidney size is clinically significant in children because small kidneys, defined as 2 SDs below the mean (5), are associated with accelerated kidney function decline in premature children (6), and kidney enlargement (2 SDs above the mean) can be observed in infiltrative kidney disease and polycystic kidney disease, among others (7,8). In the setting of a solitary functioning kidney, contralateral growth (2 SDs above the mean) is the key marker of expected compensatory growth (8).

Kidney size guidelines have been previously been based on sample sizes of less than 1800 children from birth to 18 years (9–18). A normative kidney length value chart from Rosenbaum et al in 1984 is still in use (10). Rosenbaum and colleagues’ study was performed with a small sample size (203 US examinations) and lacked sample demographic details. Subsequent studies evolved to MrNomogram (19), which requires data entry on the patient’s age, sex, race, height, and weight (19); however, these variables are not always readily available, and the model has not been studied with single-variable input.

Building on these limitations, we previously leveraged a big data approach, with radiology reports as the clinical dataset (20,21). Radiology reports are a conglomerate of unstructured data in which kidney measurements are provided (22). Natural language processing (NLP) is a computer-based approach that mines data and extracts key unstructured information from reports using pattern matching and linguistic analysis, a well-studied approach (22–24). When combined with electronic medical record searches, NLP accelerates cohort identification, enables larger sample collection with more diverse representation, reduces human abstraction errors, and potentiates research reproducibility across populations within short time frames (23–25). The aim of this study was to calculate new pediatric age-specific normative values and percentiles for kidney length and volume (17,18) through the use of an NLP model.

## Materials and Methods

### Study Sample and Data Collection

This cross-sectional study was performed at a tertiary referral center in the United States. The study received institutional review board approval with exemption from Health Insurance Portability and Accountability Act authorization to retrieve all consecutive reports from pediatric US examinations performed from January 2012 to December 2022 via the institutional imaging repository Illuminate InSight (Softek Illuminate). An advanced search was made with the terms “kidney,” “renal,” or “abdomen.” Kidney pathology terms were excluded in the search (see “renal pathologies” in Table 1).

**Table 1:** Regular Expression Terms Used for Exclusion and Kidney Measurements

Variable	Regular Expression
Renal pathologies	“dysplastic” OR “dilation” OR “dilatation” OR “duplicate” OR “horseshoe” OR “ectopia” OR “ectopic” OR “duplex” OR “solitary” OR “fused” OR “obstruction” OR “hydroureteronephrosis” OR “duplicated” OR “malrotated” OR “malignant” OR “asymmetry” OR “asymmetric” OR “scarring” OR “calculi” OR “hypoplastic” OR “hypoplasia” OR “caliectasis” OR “pelviectasis” OR “lesion” OR “ureterectasis” OR “cyst” OR “cystic” OR “pyelonephritis” OR “atrophic” OR “dysplasia” OR “transplant” OR “nephrolithiasis” OR “hydronephrosis” OR “small” OR “calculus” OR “pelvicaliectasis” OR “polycystic” OR “diverticula” OR “stones” OR “stone” OR “pelvocaliectasis” OR “hypertrophy” OR “transplanted” OR “diverticulum” OR “malrotation” OR “duplication” OR “rotation” OR “nephrectomy” OR “atrophy” OR “atrophic” OR “UTD” OR “scar” OR “abnormally” OR “absent” OR multicystic”
Urinary tract dilatation	“dilatation,” “hydronephrosis,” “UTD,” “p1,” “p2,” “p3”
Prematurity	“preterm,” “preterm,” “#w#d,” “w d/7,” “ex #w#d,” “#wker,” “h/o prematurity,” “prematurity,” “#week old”, “ex #weeker,” “ex #week infant,” “#week,” “#wk”
Kidney measurements right kidney	r"RIGHT KIDNEY:\s*(.*?)\s*,”
	r“RIGHT KIDNEY:\s*(.*?)\s*,”
	r“R KIDNEY:\s*(.*?)\s*,”
	r“RT KIDNEY:\s*(.*?)\s*,”
	r“RK:\s*(.*?)\s*,”
	r“RIGHT KIDNEY:\s*(.*?)\s*cm”
	r“RIGHT KIDNEY:\s*(.*?)\s*cm”
	r“R KIDNEY:\s*(.*?)\s*cm”
	r“RT KIDNEY:\s*(.*?)\s*cm”
	r“RK:\s*(.*?)\s*cm”
Kidney measurements left kidney	r“LEFT KIDNEY:\s*(.*?)\s*,”
	r“LEFT KIDNEY:\s*(.*?)\s*,”
	r“L KIDNEY:\s*(.*?)\s*,”
	r“LT KIDNEY:\s*(.*?)\s*,”
	r“LK:\s*(.*?)\s*,”
	r“LEFT KIDNEY:\s*(.*?)\s*cm”
	r“LEFT KIDNEY:\s*(.*?)\s*cm”
	r“L KIDNEY:\s*(.*?)\s*cm”
	r“LT KIDNEY:\s*(.*?)\s*cm”
	r"LK:\s*(.*?)\s*cm”

Note.—p1 = postnatal 1, UTD = urinary tract dilatation.

US report inclusion criteria were as follows: *(a) *reports from children from birth to 18 years and *(b)* reports from US of the kidney and bladder; retroperitoneum; Doppler renal arteries and veins; kidney limited; abdomen right upper quadrant-gallbladder, liver, and pancreas only; abdomen complete; kidney and bladder voiding with intravesical contrast; and kidney with intravenous contrast.

**Figure 1:**
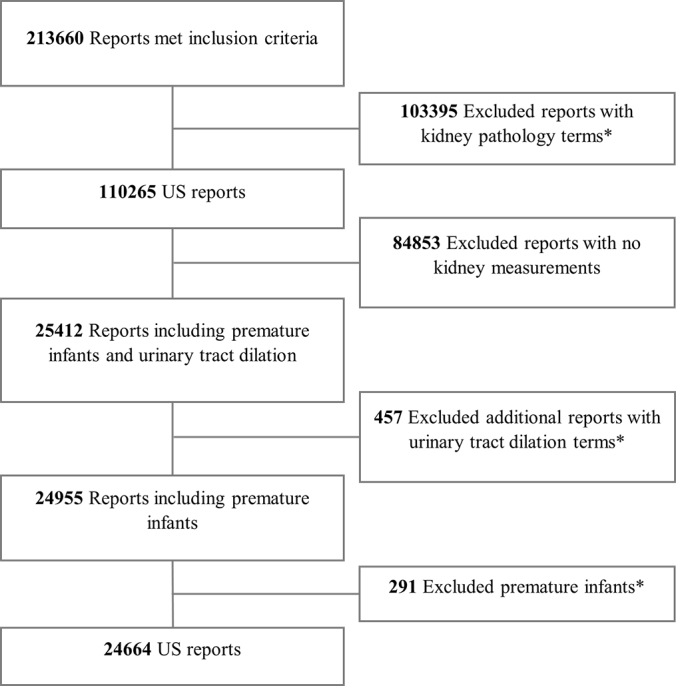
CONSORT (Consolidated Standards of Reporting Trials) diagram defining study sample. *Pathology terms, additional urinary tract dilatation terms, and prematurity terms are defined in Table 1.

Exclusion criteria are shown in Figure 1. We excluded reports that did not have kidney measurements; reports that had kidney pathologic terms; and reports from premature children, provided that the report itself specified prematurity (gestational age < 37 weeks) and related terms anywhere in the report. Sex and age information was obtained from the report search. Reports without sex information were included, with sex categorized as “unknown." Although exclusion criteria were applied using NLP, the performance of NLP was tested for correctly excluding ineligible patients. Exclusion terms were searched throughout the entire radiology report (ie, indication, findings, and impression). Two internal tests were conducted in which remaining premature and urinary tract dilatation–related terms were identified, prompting NLP model optimization using expanded regular expression terms (Table 1).

### Imaging Procedures

US examinations were performed following standardized institutional imaging protocols during the study period (US systems used and further acquisition details are available in Table S1). All examinations included multiplanar imaging of the kidneys and, when indicated, abdominal structures, with kidney measurements obtained in the sagittal or coronal plane along the longest axis. Length measurements were obtained from the upper to the lower pole. The reported kidney length represents the longest measured by the sonographer, as recommended in previous studies (10,16,19), typically in the supine position. Although institutional scanning protocols evolved over time and three standardized report templates were used during the study period, maximum kidney measurements and calculated volumes remained consistently included. Protocol updates, such as three-dimensional kidney stone measurements, postvoid imaging in infants, and prone imaging for urinary tract dilatation, did not prompt kidney metric reporting changes. Despite advances in US systems, measurements are anatomically based, and all US equipment required to meet regulatory accuracy standards is commercially available (26). The lack of change in basic measurement techniques over time validates the use of older data still used as reference standards, highlighting their continued clinical and research relevance (27), despite previously reported intraobserver and interobserver variability studies (28,29). All measurements were taken by certified pediatric sonographers. During this period, 70 sonographers were involved, 24 for 5 years or more and 46 for 1–4 years.

### Extraction of Kidney Measurements Using NLP

To extract kidney measurements from the textual data, Python programming language (version 3.9.16) and Regular Expressions (regex) Module Version 2.2.1 were used (Table 1). The kidney length abstracted was the single longest measurement reported. Kidney volume was calculated using the provided measurements in the reports, regardless of the reported volume. Calculations were performed with the ellipsoid formula: volume = length × width × height × 0.52 (30).

### Other Variables

Demographic details and anthropometric measurements (weight, height, body mass index [BMI]) were obtained from the electronic medical record search using EPIC (Epic Systems). Body measurements recorded within 31 days of the US report for children younger than 1 year, and within 90 days for children older than 1 year, were included. All measurements were entered by medical staff as part of routine clinical documentation within the electronic medical record. Height percentiles were calculated using the Centers for Disease Control and Prevention growth charts (31), and body surface area (BSA) was calculated using the Mosteller formula (32).

### Statistical Analysis

First, quantile regressions were used to determine whether median kidney length and volume differed by left versus right side, after controlling for age. Bootstrapped standard errors (SEs) were calculated to account for multiple measurements from the same individual (33). The observed difference between left and right kidneys was deemed not clinically significant for this study, as in previous studies (10,17). Hence, the mean of the available measurements (single measurement for each kidney) was calculated to provide a single kidney length and volume value per US report for subsequent analyses. If only one kidney was measured, that measurement was used as the representative value for that report.

Given that the normative values of Rosenbaum et al continue to be used as a reference standard in clinical settings at the study institution and other institutions, and that these values are still used as comparators in international studies (16,27,34), the mean kidney lengths in the current study were compared with those of the previous studies by performing linear regression with cluster-robust SEs (35) for each age group, with statistical significance assessed at the family-wise error rate of 5% (α = .05). Specifically, after adjustment for multiple comparisons using Bonferroni correction, significance was determined using a level of α/21 = .002. To compare precision, relative efficiency was calculated by dividing the SEs of this study’s estimates by the SEs of Rosenbaum and colleagues’ estimates. SEs were calculated by dividing SDs by the square root of the sample size. In addition, this study’s kidney length and volume median values were compared with Obrycki and colleagues’ (17) lamda-mu-sigma (LMS)–calculated medians using quantile regression with cluster-robust SEs for each age and sex group (36). For these comparisons, significance was determined using the Bonferroni-adjusted threshold of α/36 = .001.

New normative values for kidney length and volume were established by age group (per year) from 1 to younger than 18 years using this study’s data. Additional categories were created for children younger than 1 year (0–<1 week, 1 week–<4 months, 4–<8 months, and 8 months–<1 year) because of the increased variability in kidney growth during this period (13). The LMS method was used to create smooth centile growth curves and calculate standardized scores. This method is valuable for constructing growth curves when measurements exhibit skewed distributions (36). Age-related normal percentile curves were calculated for length and volume at the third, fifth, 10th, 25th, 50th, 75th, 90th, 95th, and 97th percentiles, which match the Centers for Disease Control and Prevention charts (34). Calibration values were calculated to assess model fit.

Among patients for whom sex and anthropometric measurements were available, quantile regression models were used to assess independent effects (16) of sex and anthropometric measurements on quantiles of kidney length and volume, after adjustment for age. Separate models were used for each anthropometric measurement (weight plus height percentile, BMI, or BSA). Raw height measurements were not included in models because of collinearity with age. Bootstrapped SEs were also calculated to account for repeated measures within individuals (33). The R^1^ goodness-of-fit measure was used to compare across models. The LMS method was also used to construct kidney volume percentile curves by BSA, with all BSAs greater than 2.5 m^2^ truncated at 2.5 m^2^. All analyses were performed by authors (J.Z. and J.L.) using R software (version 2023.12.1.402; R Core Team), and the STROBE (Strengthening the Reporting of Observational Studies in Epidemiology) cross-sectional reporting guidelines were followed to ensure study quality (37).

## Results

From the initial search, 213 660 reports were identified. A total of 188 996 were excluded: 103 395 had kidney pathologic terms, 84 853 had no kidney measurements, 291 were identified as reports from premature infants, and 457 were excluded after testing of NLP performance for urinary tract dilatation terms. The final sample included 24 664 reports from 18 769 individuals (Fig 1). A total of 3103 children had more than one report (two US reports: 1934; three US reports: 591; more than three US reports: 578). Among the 18 769 individuals, 10 134 (54%) were female, and 8185 were male; sex was not specified for 450 children (2%) (Table 2). Race and ethnicity by age group are described in Table 2 and Table S2. The sample median age was 7 years (IQR, 11 years).

**Table 2:** Demographic Characteristics of Study Patients

Characteristic	Total (*n* = 18 769)	With Anthropometric Measurements (*n* = 15 891)
Male	8183 (43.6)	7259 (45.7)
Female	10 136 (54.0)	8285 (52.1)
Unknown	450 (2.4)	247 (2.2)
Median age (IQR); range (y)	7 (11); 0–17	8 (11); 0–17
Race*		
Asian	901 (4.8)	791 (5.0)
Black or African American	3805 (20.3)	3116 (19.6)
Native American or Alaska Native	19 (0.1)	17 (0.1)
Native Hawaiian or other Pacific Islander	10 (0.05)	8 (0.1)
Other/multiracial/unknown^†^	4316 (23)	3218 (20.3)
White	9718 (51.8)	8741 (55.1)
Ethnicity		
Hispanic or Latino	2441 (13)	2095 (13.2)
Not Hispanic or Latino	15 425 (82.2)	13 619 (85.7)
Unknown^‡^	903 (4.8)	177 (1.1)
Median anthropometric measurements (IQR)		
Height (cm)	NA	125 (64.3)
Weight (kg)	NA	26.8 (38.9)
BMI	NA	17.9 (6.0)
BSA (m^2^)	NA	0.97 (0.93)

Note.—Unless otherwise noted, values are expressed as numbers, with percentages in parentheses. BMI = body mass index (calculated as weight in kilograms divided by height in meters squared), BSA = body surface area, NA = not applicable.

* Race classification was obtained from the electronic medical records.

^†^ “Other” refers to the patient-selected category in the electronic medical record.

^‡^ "Unknown" means chose not to disclose or not available.

### Right versus Left Kidney

Age-adjusted median kidney length and volume differed between the left and right sides (β = –0.09 [*P* < .001] and β = –0.53 [*P* =.01], respectively). However, these differences were deemed not clinically significant because the difference for kidney length was only 0.09 cm and that for volume was 0.53 cm^3^; these are smaller differences than those deemed not clinically significant by Rosenbaum et al (0.19 cm for length) (10) and Obrycki et al (0.103 cm for length and 2.2 cm^3^ for volume) (16,18). Therefore, the decision was made to average left and right kidney lengths and volumes for subsequent analyses.

### Comparison with Previous Normative Values

Comparisons with Rosenbaum and colleagues’ mean measurements (10) showed differences for ages 0–3 years (mean: 4.3 cm ± 0.5 to 7.3 ± 0.9 vs Rosenbaum mean: 4.5 cm ± 0.3 to 7.4 ± 0.5; *P *< .001), 4–6 years (7.6 cm ± 0.8 to 7.9 cm ± 0.9 vs 7.9 cm ± 0.5 to 8.1 cm ± 0.5; *P *< .001), 8–10 years (8.7 cm ± 0.9 to 9.0 cm ± 1.1 vs 8.9 cm ± 0.9 to 9.2 cm ± 0.9; *P* < .001), 12 years (10.0 cm ± 1.2 vs 10.4 cm ± 0.9; *P *< .001), and 15 years (10.5 cm ± 1.3 vs 10.9 cm ± 0.8; *P *< .001); for those age groups, the current study’s means were smaller. For ages 6 years (8.1 cm ± 1.0 vs 7.8 cm ± 0.7; *P *< .001), 13–15 years (10.2 cm ± 1.3 to 10.3 cm ± 1.2 vs 9.8 cm ± 0.8 to 10.1 cm ± 0.6; *P *< .001), and 16 years (10.6 cm ± 1.2 vs 10.0 cm ± 0.9; *P *< .001), the current study’s means were larger. When we compared this study’s SEs with those of Rosenbaum et al, this study’s SEs were consistently lower, ranging between 10% and 30% of Rosenbaum and colleagues’ SEs. Comparison of Obrycki and colleagues’ (17) median lengths and those of this study by age group and sex revealed statistically significant differences across most age groups in boys, with median lengths being generally shorter in this study; in girls, most differences were not significant. Volume median comparisons showed statistically significant differences in most age groups in girls and boys, with those in this study being generally larger. The IQRs of this study were larger than those of Obrycki and colleagues’ study.

### Normative Values and Percentiles

**Figure 2:**
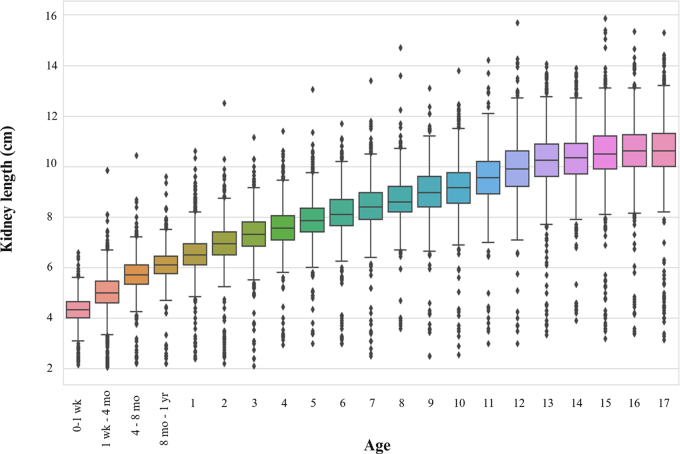
Normative kidney length values per age group with median by age ± 25% (boxes) and the fifth–95th percentile (whiskers).

**Figure 3:**
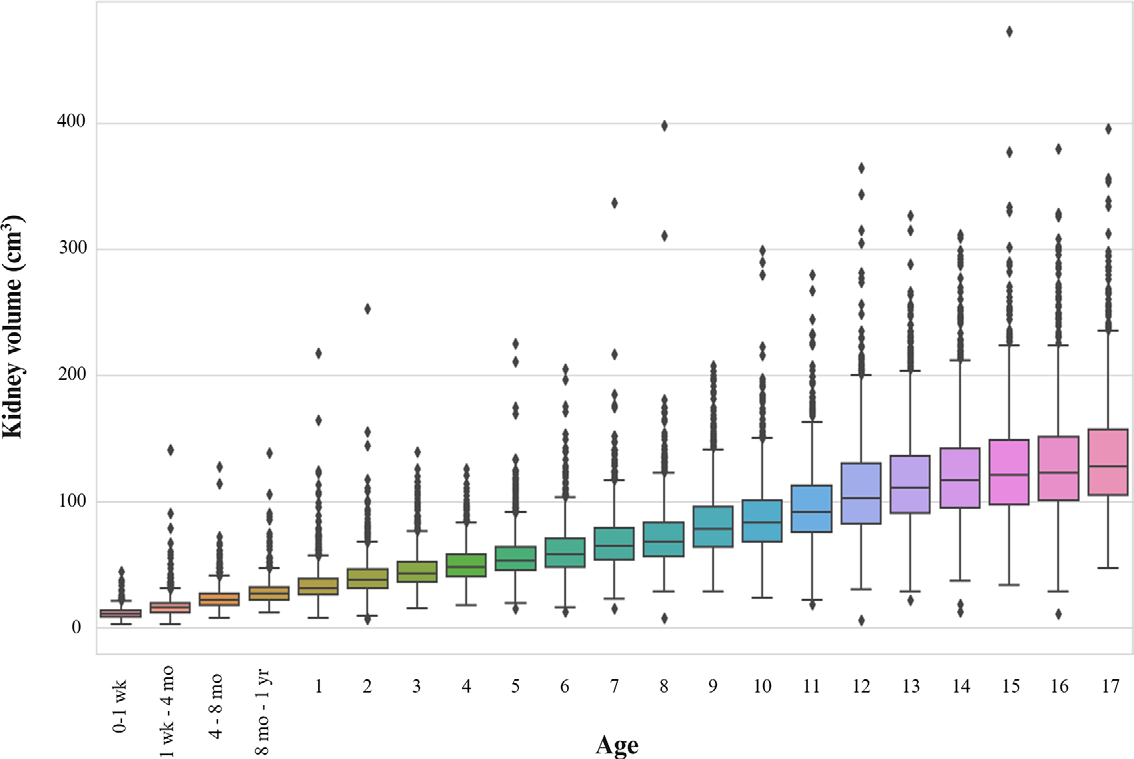
Normative kidney volume values per age group with median by age ± 25% (boxes) and the fifth–95th percentile (whiskers).

Normative kidney length and volume values per age for this study are shown in Tables 3 and 4 and Figures 2 and 3. Growth rates were highest in the 0–12-month group, in which there was a 1.8-cm change, followed by a 1.3-cm change in the 1–2-year age group and later a 0.8-cm change in the 11–13-year age group.

**Table 3:** Normative Kidney Length per Age Group

Age*	No. of Patients	Mean Length ± SD (cm)	Median Length (IQR) (cm)	Difference between Means (cm)^†^
0–<1 wk	895	4.32 ± 0.54	4.35 (0.65)	NA
1 wk–<4 mos	1708	4.99 ± 0.80	5 (0.85)	0.67
4–<8 mo	1092	5.71 ± 0.73	5.7 (0.75)	0.72
8 mo–<1 y	618	6.09 ± 0.80	6.1 (0.7)	0.38
1 y	1608	6.53 ± 0.82	6.5 (0.85)	1.28
2 y	1449	6.94 ± 0.87	6.95 (0.9)	0.41
3 y	1399	7.31 ± 0.84	7.3 (0.95)	0.37
4 y	1196	7.59 ± 0.84	7.55 (0.95)	0.28
5 y	1147	7.89 ± 0.89	7.85 (0.95)	0.3
6 y	1192	8.14 ± 0.95	8.1 (1.05)	0.25
7 y	1038	8.39 ± 1.04	8.4 (1.05)	0.25
8 y	989	8.67 ± 0.94	8.6 (1)	0.28
9 y	1038	8.97 ± 1.05	8.95 (1.2)	0.3
10 y	957	9.16 ± 1.13	9.15 (1.2)	0.19
11 y	929	9.52 ± 1.11	9.55 (1.3)	0.36
12 y	995	9.95 ± 1.24	9.9 (1.4)	0.43
13 y	1003	10.20 ± 1.30	10.25 (1.28)	0.25
14 y	1129	10.31 ± 1.17	10.35 (1.2)	0.11
15 y	1272	10.47 ± 1.29	10.5 (1.3)	0.16
16 y	1489	10.58 ± 1.18	10.6 (1.25)	0.11
17 y	1521	10.59 ± 1.28	10.6 (1.3)	0.01
Total	24 664	8.2 ± 2.15	8.25 (3.25)	5.34^‡^

Note.—NA = not available.

* Year stated represents age at time of US report and is grouped up until following age group (eg, 1 includes 1 through ≤ 2; 2 includes 2 through ≤ 3).

^†^ The shown value is the subtraction of the mean kidney length (for that age group) from the mean kidney length of the age group below.

^‡^ The difference between the kidney length mean value of the oldest and the youngest group.

**Table 4:** Normative Kidney Volume Values per Age Group

Age*	No. of Patients	Mean Volume ± SD (cm^3^)	Median Volume (IQR) (cm^3^)	Difference between Means (cm^3^)^†^
0–<1 wk	895	11.51 ± 5.46	10.95 (5.08)	NA
1 wk–<4 mo	1708	16.95 ± 14.72	15.6 (7.49)	5.44
4–<8 mo	1092	23.49 ± 9.28	22.04 (9.27)	6.54
8 mo–<1 y	618	28.35 ± 11.28	26.42 (10.31)	4.86
1 y	1608	33.95 ± 15.71	31.31 (12.71)	14.84
2 y	1449	39.7 ± 14.34	37.3 (14.76)	5.75
3 y	1399	45.36 ± 14.4	43.15 (16.43)	5.66
4 y	1196	49.98 ± 14.16	47.94 (17.38)	4.62
5 y	1147	56.47 ± 19.15	53.08 (18.82)	6.49
6 y	1192	61.11 ± 19.65	57.7 (22.22)	4.64
7 y	1038	68.19 ± 24.02	64.68 (25.38)	7.08
8 y	989	72.54 ± 26.38	68.01 (26.7)	4.35
9 y	1038	82.5 ± 27.4	78.3 (31.86)	9.96
10 y	957	87.41 ± 30	82.72 (33.22)	4.91
11 y	929	96.97 ± 32.4	91.73 (37.03)	9.56
12 y	995	110.32 ± 40.57	102.68 (47.5)	13.35
13 y	1003	117.79 ± 40.18	110.77 (45.45)	7.47
14 y	1129	121.89 ± 40.88	116.94 (47.25)	4.1
15 y	1272	127.12 ± 42.68	121.21 (51.31)	5.23
16 y	1489	129.29 ± 41.88	122.39 (50.08)	2.17
17 y	1521	134.86 ± 43.05	127.38 (51.97)	5.57
Total	24 664	72.26 ± 49.14	61.21 (67.49)	115.75^‡^

Note.—Volume was calculated using ellipsoid formula: (volume = length × width × height × 0.5233). NA = not available.

* Year stated represents age at time of US and is grouped up until following age group (eg, 1 includes 1 through ≤ 2; 2 includes 2 through ≤ 3).

^†^ The shown value is the subtraction of the mean kidney volume (for that age group) from the mean kidney volume of the age group below.

^‡^ The difference between the kidney volume mean value of the oldest and the youngest group.

For children younger than 1 year, 4313 of 24 664 (18%) reports were identified. The greatest increase in length occurred from the 1st week of life to 8 months, and a smaller increase occurred between 8 and 12 months (Table 3, Fig 2). The kidney volume change was also most pronounced during the 1st year of life and from 12 to 13 years of age (Table 4 and Fig 3).

**Figure 4:**
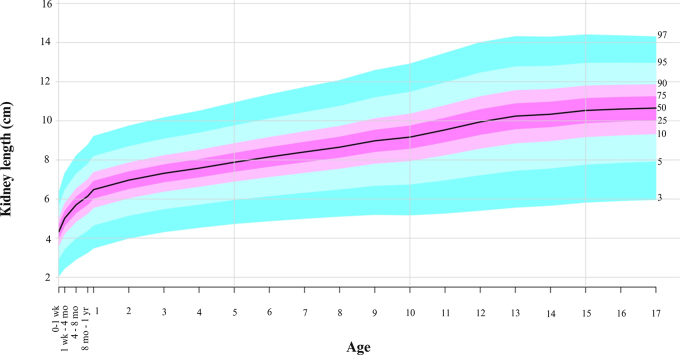
Kidney length growth curves with percentiles using the lambda-mu-sigma method; 50th percentile corresponds to the black line.

**Figure 5:**
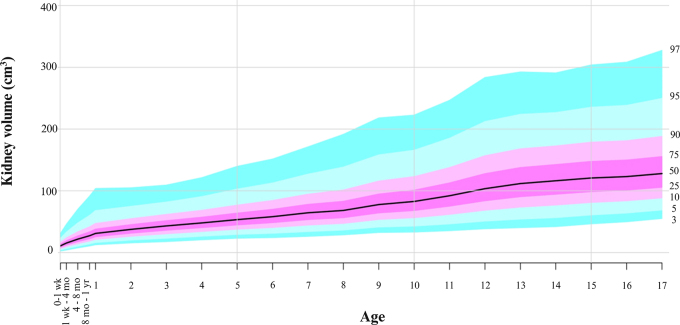
Kidney volume growth curves with percentiles using the lambda-mu-sigma method by age; 50th percentile corresponds to the black line.

The age-percentile curves for kidney length and volume are displayed in Figures 4 and 5, respectively. The LMS length and volume percentile estimated values by age are shown in Tables S3 and S4. The LMS-predicted values (for length, third percentile was 4.0 and 97th percentile was 97.2; for volume, the values were 3.1 and 97.1, respectively) closely matched the observed values across percentiles (for length, third percentile was 3.1 and 97th percentile was 97; for volume, the values were three and 97, respectively [see Table S5]), suggesting that the LMS model had good calibration and fit the data well. Percentile values by LMS for females and males are provided separately in Tables S6a, S6b, S7a, and S7b.

### Quantile Regression Models of Body Size Measurements

Reports for 1918 children age 0 to 12 months and 13 973 children age 1 to 18 years had available anthropometric measurements, totaling 15 891 reports from 12 595 children. The demographic characteristics and body size measurements are presented in Table S8. In 347 reports, sex was not described ("unknown"); therefore, 15 544 reports were used. Quantile regression models showed that weight plus height percentiles, BMI, and BSA were statistically significantly associated with median kidney length, even after adjusting for age (Table S9a). However, the magnitude of the associations was not clinically significant. For example, with age held constant, every 10-kg increase in weight or 10% increase in height was associated with a 0.1-cm higher median kidney length. Similarly, male sex was associated with an up to 0.06-cm greater median kidney length than in female patients. Therefore, LMS curves were not further stratified by sex or anthropometric measurements.

**Figure 6:**
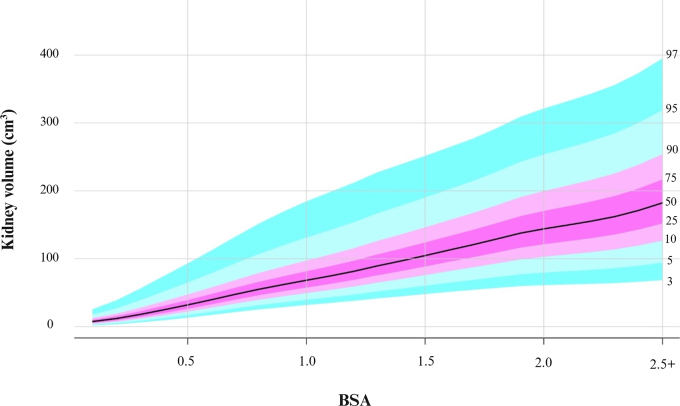
Kidney volume growth curves with percentiles using the lambda-mu-sigma method by body surface area (in meters squared).

For volume, quantile regression models showed that BMI was significantly associated with median kidney volume after adjustment for age. However, to our knowledge, reports defining a clinically significant cutoff value for kidney volume in healthy children are lacking in the literature. Therefore, significance in this study was defined as any difference greater than 5.8 cm^3^, which is the mean of the difference between the age group medians (Table 3). With age held constant, every 1-kg/m^2^ increase in BMI was associated with only a 2.0-cm^3^ greater median kidney volume (Table S9b). Similarly, every 10% increase in height was associated with only a 1.0-cm^3^ higher median kidney volume after controlling for age, sex, and weight, and male sex was associated with only up to a 3.8-cm^3^ higher median kidney volume than female sex. In contrast, median kidney volume increased by 8.9 cm^3^ for every 10-kg increase in weight (β = 8.9; *P *< .001) or 6.9 cm^3^ for every 0.1-m^2^ increase in BSA (β = 6.9; *P *< .001) respectively, which suggests clinical relevance even after controlling for age. R^1^ values were then compared, and the model with age, sex, and BSA had better fit than the model with age, sex, and weight plus height percentile (R^1^ = 0.57 vs 0.56). Furthermore, a model with BSA alone had better fit than a model with age alone (R^1^ = 0.57 vs 0.48). Therefore, LMS curves for kidney volume by BSA were plotted (Fig 6), and normative values of kidney volume by BSA and the associated calibration table are provided in Tables S10 and S11, respectively. Although BSA appears to be the better predictor of kidney volume, kidney volume percentiles for age are still reported because the weight and height variables might not always be available (Fig 5).

## Discussion

We describe normative US kidney values from a large pediatric sample. Our findings align with those of Obrycki et al (17), validating our big data approach to include a more heterogeneous sample of children undergoing US for various clinical indications. However, our kidney median lengths were generally shorter, with larger IQRs. Similarly, our kidney measurements were slightly smaller than those of Rosenbaum et al at most ages (10), likely because of our broader inclusion criteria and larger sample size. Larger IQRs and SDs likely reflect greater sample heterogeneity, reducing the risk of mischaracterizing patients as having too small or enlarged kidneys; the smaller SE suggests greater precision and reliability in estimates (38). Compared with Obrycki and colleagues’ study, we found the largest volume difference (12.3 cm^3^) in 13-year-old girls; similar differences were seen in prepubertal and pubertal ages when compared with Rosenbaum and colleagues’ values. These variations likely reflect physiologic growth spurts during puberty (39) for our North American sample, as shown in our percentile graphs, which might differ from Obrycki and colleagues’ central European sample.

Kidney growth is greatest during the first 2 years of life and at ages 8–10 and 14–18 years (40). In our sample, kidney growth was greater in the first 2 years and again at ages 12–13 years; the greatest increase was observed in children younger than 1 year, consistent with previous studies (2,10,13). Our infant sample showed the highest growth rate in both kidney length and volume during the first 8 months, aligning with the increase in glomerular filtration rate and previously defined growth in the first 7 months of life (11).

On the basis of additional nomograms, other researchers have proposed multivariable models incorporating characteristics, such as ethnicity, height, and weight (9). However, our goal was to use US efficiently because it is often available for use in various clinical scenarios, including assessments of nonkidney pathology. An accurate estimate of kidney size could help identify silent-onset kidney disease (41), even without anthropometric measurements available during image interpretation. Therefore, our normative values are based on age, a universally available variable. Analysis of other demographic factors, such as sex, has not revealed significant differences in abdominal organ size (15), which our quantile regression models corroborated.

The LMS method is not used in most nomograms (2,9–11,13,14), but in 2023 Obrycki et al developed age-based LMS normative values for kidney length and volume in a Polish and Lithuanian cohort (1758 and 1396 children, respectively) (17,18). Our LMS normative values exhibited a noticeable similarity. However, they found body height to be the most reliable predictor of kidney length, whereas we did not use height as a predictor because of its collinearity with age and age being more available in clinical practice. Our study findings reinforce previous growth charts (because we used a diverse and larger sample size), and we obtained normative values and LMS percentiles for kidney volume, which is the most precise measure of kidney size (1,3). We calculated volume with the ellipsoid formula to ensure consistency across reports. In addition, we provided charts for kidney volume by both age and BSA because BSA is a better predictor of kidney volume even though not always available.

Our study had limitations. First, our sample was drawn from a tertiary pediatric health care system, potentially including children with undiagnosed kidney pathologies not captured by our search. Imaging reports do not typically include prematurity details; therefore, the exclusion process for comorbidities and premature children may not be entirely accurate. Second, retrieving reports of right upper quadrant abdominal US could improve precision in estimating right kidney size. However, our analysis showed no clinically significant differences between right and left kidney sizes because length and volume coefficients were deemed too small to be considered useful, a finding consistent with previous literature (14,17). Third, multiple US reports pertained to the same patient; however, because the included reports were limited to nonpathologic kidneys, the measurements on these patients likely reflect normal physiologic growth rather than disease-related changes. Therefore, each measurement was treated independently in LMS curves, following World Health Organization methods for generating growth curves (42). For quantile regressions, bootstrapped SEs were used to account for the repeated measures within individuals. Fourth, retrospective data included both prone and supine measurements, but a very strong level of agreement in kidney dimensions acquired through both supine and prone positions has been reported, suggesting that these methods may be used interchangeably (16). The findings of our single institution study may not fully reflect variations in practice or patient samples from other settings. Nonetheless, this institution serves a demographically diverse sample across urban, suburban, and rural areas, with significant representation of Medicaid-enrolled children (43). We consider the cross-sectional design of this study, despite the inherent limitations of US (operator dependency, measurement variability, technologic distinctions across the study period) (28), to be an approximation of real-world clinical practices. Finally, although NLP enabled the extraction of a large dataset for our study, it has inherent limitations, including potential misclassification due to ambiguous phrasing or variability in report language and difficulties in detecting negation, which should be considered when interpreting our findings (22).

In conclusion, our study provides normative values of kidney length and volume in the largest pediatric sample examined to date. We aim to provide general pediatricians, radiologists, and pediatric subspecialists a reliable screening and reference guide using age, enhancing the practicality and accessibility of normative values. Our findings highlight distinct growth patterns, particularly within the 1st year of life reflecting physiologic milestones. Our study contributes novel LMS parameters for kidney size and increases precision, and our sample size, which is orders of magnitude larger than that of previous studies, validates our normative values with increased certainty. The provided growth curves can be included in imaging acquisition protocols to enable consistency in data acquisition and reporting, which can facilitate global research.

## Supplemental Files

Tables S1-S11

Conflicts of Interest
