# Supplementary material for: US-derived Pediatric Kidney Length and Volume Percentiles by Age: A Big Data Approach
Source: Radiol Artif Intell. 2025 Nov 19;8(1):e250056. doi: 10.1148/ryai.250056 (PMC12853003; doi:10.1148/ryai.250056)
Supplement: Tables S1-S11 [file ryai250056suppa1.pdf]

Supplemental materials undergo peer review but are not copyedited, and may include typographical errors.

©RSNA, 2025
10.1148/ryai.250056

Table S1: Ultrasound systems and acquisition details for ultrasound examinations

Manufacturer	System Models	Transducers*	Frequency Range (MHz)	Use
Philips Healthcare (Cambridge, MA, USA)	Epiq 7 to Epiq Elite	Curved, linear	1-15	Abdomen complete, RUQ/gallbladder/liver/pancreas, kidneys and bladder, renal artery-vein Doppler, kidney and bladder voiding with intravesical contrast; and kidney with intravenous contrast¥
GE Healthcare (Chicago, IL, USA)	Logiq E9 to Logiq E10	Curved, linear	1-12	Abdomen complete, RUQ/gallbladder/liver/pancreas, kidneys and bladder, renal artery-vein Doppler
Siemens Healthineers (Erlangen, Germany)	Sequoia	Curved, linear	1-12	Abdomen complete, RUQ/gallbladder/liver/pancreas, kidneys and bladder, renal artery-vein Doppler, kidney and bladder voiding with intravesical contrast; and kidney with intravenous contrast¥

* Transducer selection was age and body habitus dependent. ¥Contrast agent used: Lumason (sulfur hexafluoride lipid-type A microspheres, Bracco Diagnostics Inc, Princeton, NJ).

Supplemental materials undergo peer review but are not copyedited, and may include typographical errors.

Table S2: Demographic characteristics of study participants

**Parameter**	**Age (years)**																		
	**<1**	**1**	**2**	**3**	**4**	**5**	**6**	**7**	**8**	**9**	**10**	**11**	**12**	**13**	**14**	**15**	**16**	**17**	**Total**
n	3565	1061	893	887	795	774	852	785	766	794	745	750	792	794	931	1039	1264	1282	18769
Male* n (%)	1587 (44.5)	485 (45.7)	375 (42)	392 (44.2)	339 (42.6)	351 (45.4)	383 (45)	333 (42.4)	344 (45)	366 (46.1)	357 (47.9)	373 (49.7)	389 (49.1)	353 (44.5)	395 (42.4)	379 (36.5)	478 (37.8)	504 (39.3)	8183 (43,6)
**Race: n (%)**																			
Asian	181 (5.1)	55 (5.2)	52 (5.8)	42 (4.7)	42 (5.3)	56 (7.2)	54 (6.3)	44 (5.6)	41 (5.4)	39 (4.9)	30 (4)	42 (5.6)	38 (4.8)	29 (3.7)	36 (3.9)	32 (3.1)	47 (3.7)	41 (3.2)	901 (4.8)
Black or African American	617 (17.3)	176 (16.6)	142 (16)	143 (16.1)	144 (18.1)	147 (19)	151 (17.7)	151 (19.2)	159 (20.8)	178 (22.4)	155 (20.8)	171 (22.8)	167 (21.1)	172 (21.7)	186 (20)	258 (24.8)	305 (24.1)	383 (29.9)	3805 (20.3)
Native American or Alaska Native	4 (0.1)	1 (0.1)	1 (0.1)	1 (0.1)	-	-	1 (0.1)	2 (0.3)	-	-	1 (0.1)	2 (0.3)	1 (0.1)	1 (0.1)	1 (0.1)	-	1 (0.1)	2 (0.2)	19 (0.1)
Native Hawaiian or Other Pacific Islander	3 (0.1)	1 (0.1)	1 (0.1)	-	-	-	1 (0.1)	2 (0.3)	-	-	-	-	-	-	1 (0.1)	1 (0.1)	-	-	10 (0.05)
Other/Multiracial/ Unknown†	980 (27.5)	216 (20.4)	170 (19)	215 (24.2)	182 (22.9)	180 (23.3)	198 (23.2)	194 (24.7)	175 (22.9)	177 (22.3)	176 (23.6)	150 (20)	183 (23.1)	189 (23.8)	223 (24)	192 (18.5)	265 (21)	251 (19.6)	4316 (23)
White	1780 (49.9)	612 (57.7)	527 (59)	486 (54.8)	427 (53.7)	391 (50.5)	447 (52.5)	392 (50)	391 (51)	400 (50.4)	383 (51.4)	385 (51.3)	403 (50.1)	403 (50.8)	484 (52)	556 (53.5)	646 (51.1)	605 (47.2)	9718 (51.8)
**Ethnicity: n (%)**																			
Hispanic or Latino	517 (14.5)	140 (13.2)	107 (12)	108 (12.1)	118 (14.8)	109 (14.1)	119 (14)	112 (14.3)	115 (15)	97 (12.2)	97 (13)	95 (12.7)	109 (13.8)	94 (11.8)	128 (13.8)	120 (11.6)	130 (10.3)	126 (9.8)	2441 (13)
Not Hispanic or Latino	2789 (78.2)	897 (84.5)	770 (86.2)	757 (85.3)	665 (83.7)	639 (82.6)	694 (81.5)	646 (82.3)	628 (82)	668 (84.1)	620 (83.2)	628 (83.7)	639 (80.7)	662 (83.4)	750 (80.6)	865 (83.3)	1039 (82.2)	1069 (83.4)	15425 (81.2)
Unknown†	259 (7.3)	24 (2.3)	16 (1.8)	22 (2.5)	12 (1.5)	26 (3.4)	39 (4.6)	27 (3.4)	23 (3)	29 (3.7)	28 (3.8)	27 (3.6)	44 (5.6)	38 (4.8)	53 (5.7)	54 (5.2)	95 (7.5)	87 (6.8)	903 (4.8)

*Patients were categorized as: female, male or unknown when the report did not disclose this information.

†Chose not to disclose, not available.

Supplemental materials undergo peer review but are not copyedited, and may include typographical errors.

Table S3: Kidney length percentiles by age (LMS method)

Age (years)*	n	Percentile								
		3rd (cm)	5th (cm)	10th (cm)	25th (cm)	50th (cm)	75th (cm)	90th (cm)	95th (cm)	97th (cm)
0-<1 wk	895	3.1	3.4	3.6	4.0	4.3	4.7	5.0	5.2	5.4
1 wk-<4 mos	1708	3.7	3.9	4.2	4.6	5.0	5.4	5.8	6.1	6.3
4-<8 mos	1092	4.2	4.5	4.8	5.3	5.7	6.1	6.5	6.8	7.1
8 mos-<1 yr	618	4.6	4.9	5.3	5.7	6.2	6.6	7.0	7.3	7.6
1	1608	4.9	5.2	5.6	6.0	6.5	6.9	7.4	7.7	8.0
2	1449	5.4	5.7	6.1	6.5	7.0	7.4	7.9	8.2	8.5
3	1399	5.7	6.0	6.4	6.9	7.3	7.8	8.2	8.6	8.8
4	1196	6.0	6.3	6.6	7.1	7.6	8.0	8.5	8.9	9.1
5	1147	6.2	6.5	6.9	7.4	7.9	8.3	8.8	9.2	9.5
6	1192	6.4	6.7	7.1	7.7	8.2	8.6	9.2	9.5	9.9
7	1038	6.6	6.9	7.4	7.9	8.4	8.9	9.4	9.9	10.2
8	989	6.8	7.1	7.6	8.1	8.7	9.2	9.7	10.1	10.5
9	1038	7.0	7.4	7.8	8.4	9.0	9.5	10.1	10.6	10.9
10	957	7.1	7.5	8.0	8.6	9.2	9.7	10.4	10.8	11.2
11	929	7.3	7.7	8.3	8.9	9.5	10.1	10.8	11.3	11.6
12	995	7.6	8.0	8.6	9.3	9.9	10.6	11.2	11.7	12.1
13	1003	7.9	8.3	8.9	9.6	10.2	10.9	11.5	12.0	12.4
14	1129	8.0	8.4	9.0	9.7	10.3	11.0	11.6	12.1	12.5
15	1272	8.2	8.6	9.2	9.9	10.5	11.1	11.8	12.3	12.6
16	1489	8.3	8.7	9.3	10.0	10.6	11.2	11.8	12.3	12.7
17	1521	8.3	8.8	9.3	10.0	10.7	11.2	11.9	12.3	12.7

mos: months. wk: week. yr: year.

*Year stated represents age at time of ultrasound and is grouped up until following age group (i.e., 1 = 1 ≤ 2; 2 = 2 ≤ 3, etc.).

Supplemental materials undergo peer review but are not copyedited, and may include typographical errors.

Table S4: Kidney volume percentiles by age (LMS method)

Age (years)*	n	Percentile								
		3rd (cm^3)	5th (cm^3)	10th (cm^3)	25th (cm^3)	50th (cm^3)	75th (cm^3)	90th (cm^3)	95th (cm^3)	97th (cm^3)
0-<1 wk	895	5	5	7	9	11	14	17	20	22
1 wk-<4 mos	1708	7	8	10	12	15	19	24	28	31
4-<8 mos	1092	11	12	14	18	22	27	34	39	44
8 mos-<1 yr	618	15	16	18	22	27	34	42	49	55
1	1608	17	19	21	26	31	38	47	55	62
2	1449	22	23	26	31	38	45	55	63	69
3	1399	25	27	30	36	43	52	62	70	76
4	1196	28	30	34	40	48	57	69	77	85
5	1147	31	34	38	45	53	64	77	87	96
6	1192	33	36	40	48	58	70	85	96	105
7	1038	36	39	44	53	64	78	95	108	118
8	989	39	42	47	56	68	83	101	116	128
9	1038	44	48	54	64	78	95	116	133	147
10	957	46	50	56	68	83	101	123	140	154
11	929	50	54	62	75	92	113	138	157	172
12	995	55	60	68	84	104	128	157	180	197
13	1003	58	64	74	90	112	138	168	191	209
14	1129	61	67	77	94	116	143	173	195	213
15	1272	65	71	81	99	121	148	179	202	221
16	1489	68	74	84	101	123	150	181	205	223
17	1521	73	79	88	106	128	156	188	214	233

mos: months. wk: week. yr: year.

*Year stated represents age at time of ultrasound and is grouped up until following age group (i.e., $1 = 1 \leq 2$; $2 = 2 \leq 3$, etc.).

Supplemental materials undergo peer review but are not copyedited, and may include typographical errors.

Table S5: Kidney length and volume percentiles by age: LMS calibrated values vs sample values

Percentile	Length		Volume	
	Calibration	Sample	Calibration	Sample
3rd	4.03	3.02	3.14	3
5th	6.28	5.01	5.15	5
10th	11.25	10.02	10.18	10
25th	24.48	25.02	24.60	25
50th	48.89	50.15	49.03	50
75th	75.83	74.90	75.14	75
90th	90.69	90	90.06	90
95th	95.33	95	95.03	95
97th	97.16	97	97.05	97

Supplemental materials undergo peer review but are not copyedited, and may include typographical errors.

Table S6a: Kidney length percentiles by age for females only (LMS method)

Age (years)*	n	Percentile								
		3rd (cm)	5th (cm)	10th (cm)	25th (cm)	50th (cm)	75th (cm)	90th (cm)	95th (cm)	97th (cm)
0-<1 wk	297	3.1	3.4	3.6	4.0	4.3	4.6	4.9	5.1	5.3
1 wk-<4 mos	726	3.7	3.9	4.2	4.6	5.0	5.3	5.7	6.0	6.1
4-<8 mos	526	4.3	4.5	4.9	5.3	5.7	6.1	6.5	6.7	7.0
8 mos-<1 yr	322	4.6	4.9	5.2	5.7	6.1	6.5	6.9	7.2	7.5
1	803	4.9	5.2	5.5	6.0	6.4	6.9	7.3	7.6	7.8
2	751	5.4	5.7	6.1	6.5	7.0	7.4	7.9	8.2	8.4
3	737	5.7	6.0	6.4	6.8	7.3	7.7	8.2	8.5	8.7
4	656	6.0	6.3	6.7	7.1	7.6	8.0	8.5	8.8	9.1
5	611	6.3	6.6	6.9	7.4	7.9	8.4	8.8	9.2	9.5
6	638	6.4	6.7	7.1	7.7	8.1	8.6	9.1	9.5	9.8
7	584	6.6	7.0	7.4	7.9	8.4	8.9	9.4	9.8	10.1
8	529	6.8	7.1	7.5	8.1	8.6	9.1	9.7	10.1	10.4
9	564	7.0	7.4	7.8	8.4	9.0	9.6	10.2	10.6	10.9
10	497	7.2	7.6	8.1	8.7	9.3	9.9	10.5	11.0	11.3
11	475	7.4	7.9	8.4	9.0	9.6	10.2	10.9	11.3	11.7
12	511	7.8	8.2	8.7	9.4	10.0	10.6	11.3	11.7	12.1
13	552	8.0	8.4	9.0	9.6	10.3	10.9	11.5	12.0	12.3
14	646	8.1	8.5	9.0	9.7	10.3	10.9	11.5	12.0	12.3
15	785	8.2	8.6	9.1	9.8	10.4	11.0	11.6	12.1	12.4
16	920	8.2	8.6	9.2	9.9	10.5	11.0	11.6	12.1	12.4
17	918	8.2	8.7	9.2	9.9	10.5	11.1	11.6	12.1	12.4

mos: months. wk: week. yr: year.
*Year stated represents age at time of ultrasound and is grouped up until following age group (i.e., 1 = 1 ≤ 2; 2 = 2 ≤ 3, etc.).

Supplemental materials undergo peer review but are not copyedited, and may include typographical errors.

Table S6b: Kidney length percentiles by age for males only (LMS method)

Age (years)*	n	Percentile								
		3rd (cm)	5th (cm)	10th (cm)	25th (cm)	50th (cm)	75th (cm)	90th (cm)	95th (cm)	97th (cm)
0-<1 wk	338	3.2	3.5	3.8	4.2	4.5	4.9	5.3	5.6	5.8
1 wk-<4 mos	830	3.6	3.8	4.2	4.6	5.0	5.4	5.8	6.1	6.3
4-<8 mos	480	4.2	4.5	4.9	5.3	5.7	6.2	6.6	7.0	7.2
8 mos-<1 yr	258	4.7	5.0	5.4	5.9	6.3	6.8	7.2	7.6	7.9
1	726	4.9	5.2	5.6	6.1	6.5	7.0	7.4	7.8	8.1
2	655	5.4	5.7	6.0	6.5	7.0	7.4	7.9	8.2	8.5
3	645	5.7	6.0	6.4	6.9	7.4	7.8	8.3	8.7	9.0
4	540	5.9	6.2	6.6	7.1	7.6	8.0	8.5	8.9	9.2
5	533	6.1	6.5	6.9	7.4	7.9	8.3	8.8	9.2	9.5
6	552	6.4	6.7	7.1	7.7	8.2	8.7	9.2	9.6	9.9
7	454	6.5	6.9	7.3	7.9	8.4	8.9	9.4	9.9	10.2
8	460	6.8	7.1	7.6	8.2	8.7	9.2	9.8	10.2	10.5
9	474	7.0	7.3	7.8	8.4	8.9	9.5	10.0	10.5	10.8
10	460	7.0	7.4	7.9	8.5	9.0	9.6	10.2	10.6	10.9
11	454	7.2	7.6	8.1	8.8	9.4	10.0	10.6	11.1	11.5
12	483	7.4	7.9	8.5	9.2	9.9	10.5	11.2	11.7	12.1
13	451	7.6	8.1	8.7	9.5	10.2	10.9	11.6	12.1	12.5
14	482	7.8	8.3	8.9	9.7	10.4	11.0	11.7	12.3	12.7
15	486	8.2	8.7	9.3	10.0	10.7	11.4	12.0	12.6	13.0
16	567	8.5	8.9	9.5	10.2	10.8	11.4	12.1	12.6	13.0
17	602	8.7	9.1	9.6	10.3	10.9	11.5	12.1	12.6	12.9

mos: months. wk: week. yr: year.

*Year stated represents age at time of ultrasound and is grouped up until following age group (i.e., 1 = 1 ≤ 2; 2 = 2 ≤ 3, etc.).

Supplemental materials undergo peer review but are not copyedited, and may include typographical errors.

Table S7a: Kidney volume percentiles by age for females only (LMS method)

Age (years)*	n	Percentile								
		3rd (cm^3)	5th (cm^3)	10th (cm^3)	25th (cm^3)	50th (cm^3)	75th (cm^3)	90th (cm^3)	95th (cm^3)	97th (cm^3)
0-<1 wk	297	5	5	6	8	10	13	16	18	20
1 wk-<4 mos	726	7	8	9	12	15	19	23	27	29
4-<8 mos	526	11	12	14	17	21	26	32	37	40
8 mos-<1 yr	322	14	15	17	21	26	32	39	45	49
1	803	16	18	20	25	30	37	45	51	56
2	751	21	23	26	31	37	45	54	61	67
3	737	25	27	30	36	43	51	61	69	75
4	656	27	30	33	40	47	57	68	76	83
5	611	31	33	37	44	53	64	76	86	94
6	638	33	36	40	48	57	69	83	94	102
7	584	36	39	44	53	64	78	94	106	116
8	529	38	41	46	56	68	82	100	114	124
9	564	43	46	53	64	78	96	117	133	146
10	497	45	49	56	69	84	104	127	145	160
11	475	49	54	62	75	93	115	140	161	177
12	511	53	59	67	83	102	125	153	175	192
13	552	58	64	73	89	109	133	162	184	201
14	646	61	67	76	92	112	135	163	184	201
15	785	64	69	78	94	113	137	164	184	201
16	920	66	72	80	96	115	139	166	186	202
17	918	70	76	85	100	120	144	172	193	210

mos: months. wk: week. yr: year.

*Year stated represents age at time of ultrasound and is grouped up until following age group (i.e., $1 = 1 \leq 2$; $2 = 2 \leq 3$, etc.).

Supplemental materials undergo peer review but are not copyedited, and may include typographical errors.

Table S7b: Kidney volume percentiles by age for males only (LMS method)

Age (years)*	n	Percentile								
		3rd (cm^3)	5th (cm^3)	10th (cm^3)	25th (cm^3)	50th (cm^3)	75th (cm^3)	90th (cm^3)	95th (cm^3)	97th (cm^3)
0-<1 wk	338	5	6	7	9	12	15	19	22	25
1 wk-<4 mos	830	7	8	9	12	15	19	24	28	32
4-<8 mos	480	11	12	14	18	22	28	35	41	47
8 mos-<1 yr	258	16	17	20	24	29	36	45	53	61
1	726	18	19	22	26	32	39	49	58	66
2	655	22	24	27	32	38	46	56	65	72
3	645	26	28	31	37	44	52	62	71	78
4	540	29	31	35	41	49	58	69	78	85
5	533	32	34	38	45	54	64	77	88	97
6	552	35	37	41	49	59	71	87	100	110
7	454	37	40	45	53	65	79	97	111	123
8	460	40	43	48	57	69	84	103	119	131
9	474	45	48	54	64	77	94	114	130	142
10	460	46	50	56	67	81	99	119	135	147
11	454	50	55	62	75	91	111	135	154	168
12	483	56	62	70	85	106	131	160	183	200
13	451	59	65	75	92	115	143	175	199	217
14	482	63	70	80	99	123	153	186	211	229
15	486	70	77	89	109	135	165	200	226	245
16	567	75	82	93	112	137	167	201	227	245
17	602	80	87	97	117	142	172	206	232	252

mos: months. wk: week. yr: year.

*Year stated represents age at time of ultrasound and is grouped up until following age group (i.e., $1 = 1 \leq 2$; $2 = 2 \leq 3$, etc.).

Supplemental materials undergo peer review but are not copyedited, and may include typographical errors.

Table S8: Demographic characteristics of subgroup with body size measurements

Characteristics s	Total (12,595)
Female: n (%)	6604 (52.4)*
Age (years): median (IQR)	8 (2-13)
Race: n (%)	
. Asian	631 (5)
. Black or African American	2584 (20.5)
. Native American or Alaska Native	12 (0.1)
. Native Hawaiian or Other Pacific Islander	7 (0.1)
. Other/Multiracial/Unknown†	2494 (19.8)
. White	6867 (54.5)
Ethnicity: n (%)	
. Hispanic or Latino	1671 (13.3)
. Not Hispanic or Latino	10839 (86.1)
. Unknown†	85 (0.7)
Weight (Kg): median (IQR)	12.5 (12.9-54.4)
Height (cm): median (IQR)	127 (88-157.5)
BSA Mosteller (m^2): median (IQR)	0.99 (0.56-1.55)
BMI (Kg/m^2): median (IQR)	17.9 (15.76-22.03)

*Patients were categorized as: female, male or unknown when the report did not disclose this information.
†Unknown: chose not to disclose, not available.

Supplemental materials undergo peer review but are not copyedited, and may include typographical errors.

Table S9a: Multivariable quantile regression models associated with median kidney length, adjusted for age

Model	Variable	Coefficient (SE)	p-value	R1
Model 1*	Age (years)	0.28 (0.002)	<0.001	0.53
	Sex (male)	0.06 (0.02)	<0.001	
	BMI (kg/m^2)	0.04 (0.002)	<0.001	
Model 2†	Age (years)	0.25 (0.003)	<0.001	0.57
	Sex (male)	0.04 (0.02)	.01	
	Weight (per 10 Kg)	0.11 (0.007)	<0.001	
	Height (percentiles by 10% increment)	0.10 (0.002)	<0.001	
Model 3‡	Age (years)	0.11 (0.003)	<0.001	0.57
	Sex (male)	-0.03 (0.01)	.06	
	BSA Mosteller (per 0.1 m^2)	0.21 (0.004)	<0.001	

BMI: body mass index. BSA: body surface area, SE: standard error.
*Model 1 shows that holding age constant, every 1 kg/m2 increase in BMI was only associated with 0.04 cm higher median kidney length.
†Model 2 shows that holding age constant, every 10 kg increase in weight or 10 percentile increase in height was only associated with 0.1 cm higher median kidney length.
‡ Model 3 shows that holding age constant every 0.1 m2 increase in BSA was only associated with 0.2 cm higher median kidney length.

Supplemental materials undergo peer review but are not copyedited, and may include typographical errors.

Table S9b: Multivariable quantile regression models associated with median kidney volume, adjusted for age

Model	Variable	Coefficient (SE)	p-value	R1
Model 1*	Age (years)	5.47 (0.04)	<0.001	0.52
	Sex (male)	3.77 (0.29)	<0.001	
	BMI (Kg/m2)	2.02 (0.06)	<0.001	
Model 2†	Age (years)	3.10 (0.08)	<0.001	0.56
	Sex (male)	2.78 (0.28)	.01	
	Weight (per 10 Kg)	8.93 (0.25)	<0.001	
	Height (percentiles by 10% increment)	1.02 (0.05)	<0.001	
Model 3‡	Age (years)	0.39 (0.10)	<0.001	0.57
	Sex (male)	1.82 (0.25)	<0.001	
	BSA Mosteller (per 0.1 m^2)	6.86 (0.12)	<0.001	

BMI: body mass index. BSA: body surface area. SE: standard error.

*Model 1 shows that holding age constant, every 1 kg/m2 increase in BMI was associated with 2.0 cm^3 higher median kidney volume.

†Model 2 shows that every 10 percentile increase in height was associated with 1.0 cm3 higher median kidney volume after controlling for age, sex, and weight. And for every 10kg increase in weight median kidney volume increased by 8.9 cm^3.

‡Model 3 shows that for every 0.1 increase in BSA median kidney volume increased by 6.9 cm^3.

Supplemental materials undergo peer review but are not copyedited, and may include typographical errors.

Table S10: Kidney volume percentiles by BSA (LMS method)

BSA*	Percentile								
	3rd (cm^3)	5th (cm^3)	10th (cm^3)	25th (cm^3)	50th (cm^3)	75th (cm^3)	90th (cm^3)	95th (cm^3)	97th (cm^3)
0.1	3.5	3.9	4.7	5.9	7.5	9.6	12.1	14.1	15.8
0.2	6.0	6.7	7.7	9.6	11.9	14.9	18.6	21.6	24.2
0.3	9.6	10.6	12.1	14.7	18.0	22.1	27.3	31.6	35.1
0.4	13.9	15.2	17.1	20.6	24.8	30.2	36.9	42.5	47.2
0.5	18.6	20.2	22.6	26.8	32.0	38.6	46.8	53.6	59.4
0.6	23.7	25.5	28.4	33.4	39.6	47.4	57.2	65.3	72.1
0.7	28.9	31.0	34.4	40.2	47.5	56.7	68.0	77.5	85.5
0.8	33.8	36.2	40.0	46.7	55.0	65.5	78.5	89.3	98.3
0.9	38.1	40.8	45.1	52.5	61.8	73.4	87.9	99.9	109.9
1.0	42.1	45.2	49.8	58.0	68.2	81.0	96.8	110.0	120.9
1.1	46.1	49.4	54.5	63.5	74.6	88.4	105.6	119.7	131.3
1.2	50.3	53.9	59.6	69.3	81.5	96.5	114.9	130.0	142.4
1.3	55.2	59.2	65.4	76.2	89.5	105.9	125.8	141.9	155.1
1.4	59.5	63.9	70.7	82.4	96.8	114.4	135.4	152.3	166.0
1.5	64.2	69.0	76.4	89.1	104.6	123.4	145.7	163.3	177.4
1.6	68.9	74.1	82.2	96.0	112.7	132.7	156.2	174.6	189.2
1.7	73.4	79.0	87.7	102.6	120.5	141.8	166.6	185.8	200.9
1.8	77.9	84.0	93.5	109.6	128.9	151.7	178.0	198.2	214.0
1.9	82.3	89.0	99.2	116.7	137.6	162.0	190.0	211.4	228.0
2.0	85.2	92.4	103.3	121.9	144.0	169.8	199.2	221.4	238.6
2.1	87.6	95.1	106.7	126.3	149.6	176.6	207.2	230.2	247.9
2.2	89.9	97.9	110.1	130.8	155.4	183.7	215.5	239.3	257.6
2.3	92.8	101.2	114.2	136.2	162.1	191.9	225.2	249.9	268.7
2.4	96.9	106.0	120.0	143.5	171.2	202.9	237.9	263.8	283.5
2.5	102.0	111.9	127.1	152.5	182.3	216.2	253.4	280.8	301.4

BSA, body surface area (m^2).
*BSA calculated by Mosteller formula.

Supplemental materials undergo peer review but are not copyedited, and may include typographical errors.

Table S11: Kidney length and volume percentiles by BSA: LMS calibrated values vs sample values

Percentile	Length		Volume	
	Calibration	Sample	Calibration	Sample
3rd	4.56	3.02	3.21	3.01
5th	6.96	4.94	5.25	5.00
10th	11.25	10.01	10.21	10
25th	24.52	25.00	24.58	25
50th	49.23	50.11	49.84	50
75th	75.79	75.14	75.20	75
90th	90.72	89.96	90.00	90
95th	95.31	95	94.83	95
97th	97.02	97	96.92	97
