# Supplementary material for: US-derived Pediatric Kidney Length and Volume Percentiles by Age: A Big Data Approach
Source: Radiol Artif Intell. 2025 Nov 19;8(1):e250056. doi: 10.1148/ryai.250056 (PMC12853003; doi:10.1148/ryai.250056)
Supplement: Conflicts of Interest [file ryai250056coi.zip › 527187199_93697920_1746239604097.pdf]

**ICMJE DISCLOSURE FORM**

Instructions

In the interest of transparency, we ask you to disclose all employment/relationships/activities/interests listed below that are related to the content of your manuscript. “Related” means any relationship with for-profit or not-for-profit third parties whose interests may be affected by the content of the manuscript. Disclosure represents a commitment to transparency and does not necessarily indicate a bias. If you are in doubt about whether to list an employment/relationship/activity/interest, it is preferable that you do so.

The following questions apply to your employment/relationships/activities/interests as they relate to the **current manuscript only**. Each author is required to submit a separate form and is responsible for the accuracy and completeness of the submitted information.

Your employment/relationships/activities/interests should be **defined broadly**. For example, if your manuscript pertains to the epidemiology of hypertension, you should declare all relationships with manufacturers of antihypertensive medication, even if that medication is not mentioned in the manuscript.

| | |
|---|---|
| Date: | 02-May-2025 |
| req First Name: | Summer |
| req Last Name: | Kaplan |

Manuscript Title: Ultrasound-Derived Pediatric Kidney Length and Volume Percentiles by Age: A Big Data Approach

Manuscript number: RYAI-25-0056.R1

**In item #1 below, report all support for the work reported in this manuscript without time limit. For all other items, the time frame for disclosure is the past 36 months. Note: All items #1 through #13 must indicate none (by checking the box next to None) or include relevant disclosure information in the text boxes. Blank rows will cause the form to be sent back for completion.**

| | **Name all entities with whom you have this relationship or check the box next to None** | **Specifications/Comments (e.g., if payments were made to you or to your institution)** |
|---|---|---|
| **Time frame: Since the initial planning of the work** | | |
| 1. All support for the present manuscript (e.g., funding, provision of study materials, medical writing, article processing charges, etc.) **No time limit for this item.** | ✓ | None |
| | | |

| Time frame: past 36 months | | |
|---|---|---|
| 2. Grants or contracts from any entity (if not indicated in item #1 above). | ✓ | None |
| | | |
| 3. Royalties or licenses | ✓ | None |
| | | |
| 4. Consulting fees | ✓ | None |
| | | |
| 5. Payment or honoraria for lectures, presentations, speakers bureaus, manuscript writing or educational events | ✓ | None |
| | | |
| 6. Payment for expert testimony | ✓ | None |
| | | |
| 7. Support for attending meetings and/or travel | ✓ | None |
| | | |
| 8. Patents planned, issued or pending | ✓ | None |
| | | |
| 9. Participation on a Data Safety Monitoring Board or Advisory Board | ✓ | none |
| | | |
| 10. Leadership or fiduciary role in other board, society, committee or advocacy group, paid or unpaid | ✓ | None |
| | | |
| 11. Stock or stock options | ✓ | None |
| | | |
| 12. Receipt of equipment, materials, drugs, medical writing, gifts or other services | ✓ | None |
| | | |
| 13. Other financial or non-financial interests | ✓ | None |
| | | |

req **Please check the box next to the following statement to indicate your agreement:**

✓ **I certify that I have answered every question and all the information is complete and accurate.**

*This is a reprint of the ICMJE Recommendations for the Conduct, Reporting, Editing and Publication of Scholarly Work in Medical Journals. RSNA prepared this reprint. The ICMJE has not endorsed nor approved the contents of this reprint. The official version of the Recommendations for the Conduct, Reporting, Editing and Publication of Scholarly Work in Medical Journals is located at www.ICMJE.org. Users should cite this official version when citing the document.*
